# Chronic Inguinal Pain After Kidney Transplantation, a Common and Underexposed Problem

**DOI:** 10.1007/s00268-016-3713-9

**Published:** 2016-09-16

**Authors:** Marcel Zorgdrager, Johan. F. M. Lange, Christina. Krikke, Gertrude. J. Nieuwenhuijs, Sybrand. H. Hofker, Henri. G. D. Leuvenink, Robert A. Pol

**Affiliations:** 1 Department of Surgery, Division of Transplant Surgery, University Medical Center Groningen, University of Groningen, P.O. Box 30 001, 9700 RB Groningen, The Netherlands; 2 Department of Anesthesiology, University Medical Center Groningen, University of Groningen, Groningen, The Netherlands

## Abstract

**Background:**

The incidence and impact of chronic inguinal pain after kidney transplantation is not clearly established. A high incidence of pain after inguinal hernia repair, a comparable surgical procedure, suggests an underexposed problem.

**Methods:**

Between 2011 and 2013, 403 consecutive patients who underwent kidney transplantation were invited to complete the Caroline Comfort Scale (CCS) and Visual Analog Scale (VAS) in order to assess the incidence of chronic inguinal pain and movement disabilities, complemented by questions regarding comorbidity during follow-up.

**Results:**

The response rate was 58 % (*n* = 199) with a median follow-up of 22 months (IQR 12–30). In total, 90 patients (45 %) reported a CCS > 0 and 64 patients (32 %) experienced at least mild but bothersome complaints. Most inguinal complaints were reported during bending over and walking with a mean CCS score of 1.1 (SD ± 2.2) and 1.2 (SD ± 2.4), respectively. A high body mass index (BMI), delayed graft function, and the need for a second operation were associated with a higher CCS score on univariate analysis. Using multivariate analysis, only BMI (*p* = 0.02) was considered an independent risk factor for chronic inguinal pain.

**Conclusions:**

The incidence of chronic inguinal pain is a common though underexposed complication after kidney transplantation. More awareness to prevent neuropathic pain seems indicated.

## Introduction

Kidney transplantation remains the preferred treatment in patients with end-stage renal disease and not only results in a better survival but also in an improved quality of life (QoL) [1, 2]. QoL is strongly influenced by (chronic) pain and the incidence after kidney transplantation is estimated as high as 62 % [3]. The etiology of this chronic pain, however, is largely unknown.

**Fig. 1:**
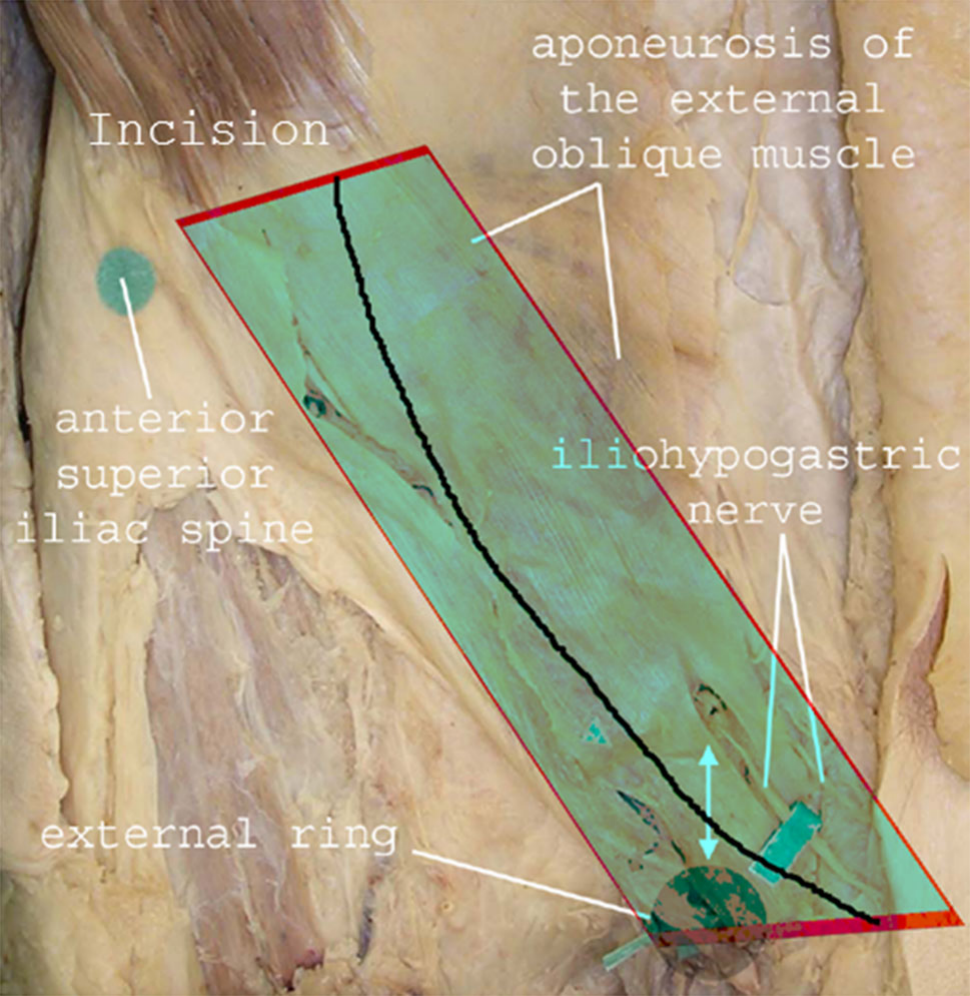
Preperitoneal or superficial surgical layer is shown. At this level, the iliohypogastic and ilioinguinal nerves are at risk. The ilioinguinal nerve is located parallel to the inguinal ligament and deep to the internal oblique aponeurosis (both not shown)

**Fig. 2:**
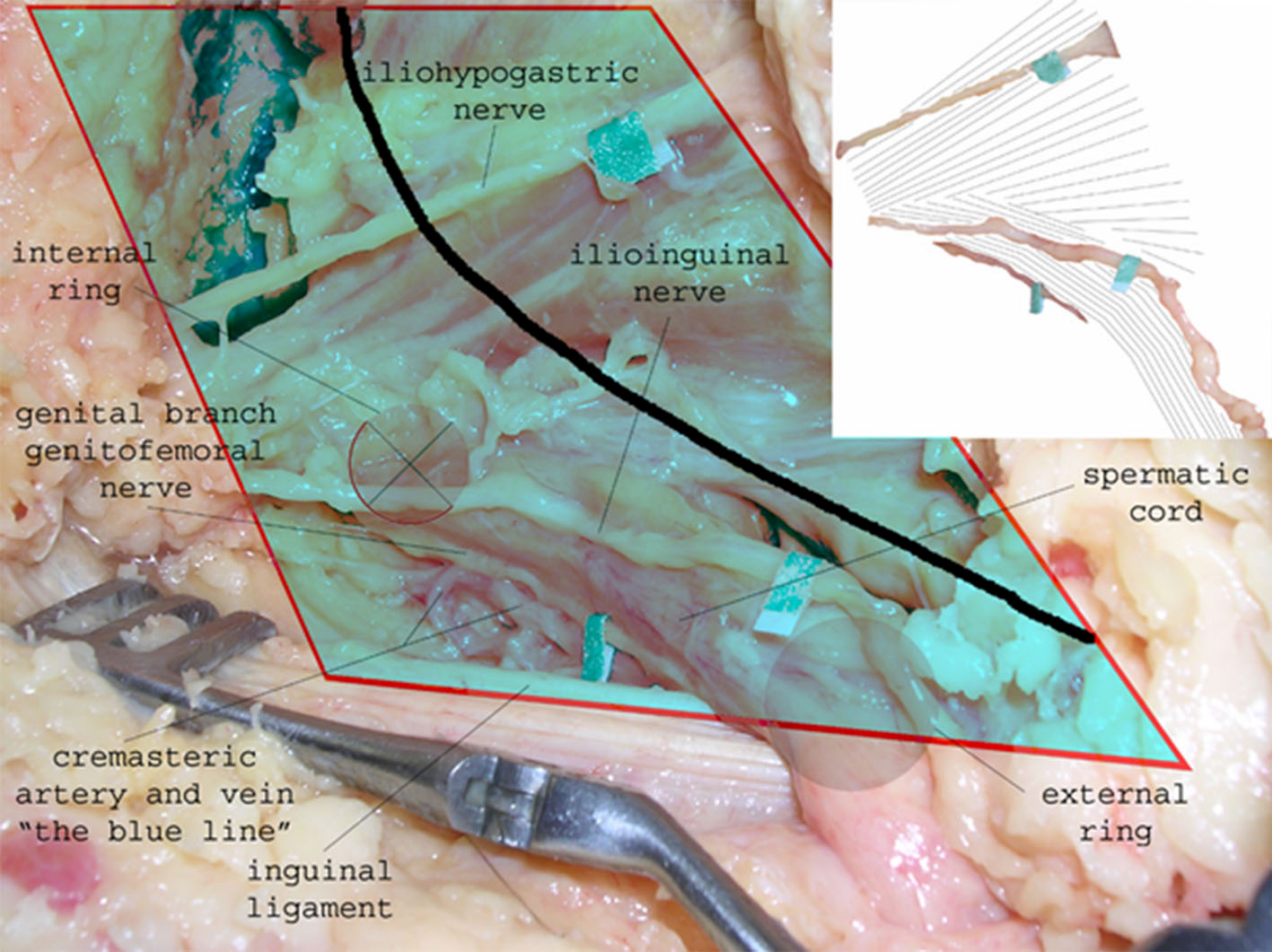
Retroperitoneal space is shown. All three nerves are identified within the preparation space and are at risk during dissection

Inguinal herniorrhaphy, a surgical procedure located in comparable anatomical levels in the inguinal region, is known for its high risk (15–53 %) of chronic pain [4]. Herein three types of chronic pain are described in the literature: somatic, neuropathic, and visceral pain [5]. Several techniques, approaches, and hypothesis have been formulated to minimize the risk of developing chronic pain. First, the type of mesh used appears to play a role in developing chronic pain after inguinal hernia repair [6]. Second, the proper identification of the three inguinal nerves appears to prevent neuropathic chronic pain [4]. During incision and dissection, the ilioinguinal and iliohypogastric nerves are first encountered in the superficial preperitoneal surgical plane (Fig. 1), and at the deep retroperitoneal level, all three nerves can be identified within the surgical space (Fig. 2). Thirdly, prophylactic neurectomy is considered an option when the risk of chronic inguinal pain is expected to be high [7].

A large Dutch study among surgeons and surgical residents showed a reduced understanding of inguinal neurologic anatomy and an underestimation of the chronic inguinal pain incidence after hernia repair [8]. Because of the comparable anatomical approach during kidney transplantation, we hypothesized that the chronic pain after transplantation has a similar etiology and is a relatively underexposed problem. To assess this theory, we performed a cohort analysis with emphasis on inguinal chronic pain in kidney transplant patients.

## Patients and methods

Between 2011 and 2013, 403 consecutive patients who underwent kidney transplantation were invited to complete the questionnaires [9]. These data were added to a large prospective database of kidney transplant patients. Exclusion criteria were combined kidney/pancreas transplantation, transplantation through a midline laparotomy, age < 18 years, incapability to fill out the questionnaire (e.g., intellectual disability or foreign language), and death during follow-up. There was no upper limit of BMI for performing kidney transplantation in our center; patients with a BMI of >35 were discussed in a multidisciplinary panel consisting of nephrologists and surgeons where the safety and technical feasibility were determined. Informed consent was obtained from all included patients.

### Transplantation procedure

An oblique surgical incision was performed from the pubic bone through about two centimeters cranial to the superior anterior iliac spine. The internal and external oblique fasciae were closed by dissolvable polydioxanone sutures, and the skin was closed by staples. Postoperative pain management consisted of paracetamol and administration of piritramide (Dipidolor^®^) intravenously or subcutaneously. A patient-controlled analgesia pump was occasionally used. Nonsteroidal anti-inflammatory drugs were avoided because of nephrotoxic side effects. The postoperative pain treatment during the first 24 h was evaluated by anesthetists.

### Clinical data selection

Collected data consisted of age, gender, body mass index (BMI; weight in kg/height in meters squared), American Society of Anaesthesiologists (ASA) score, complications according to Clavien-Dindo classification (within 30 days after transplantation), type of graft, site of transplantation, need for re-intervention, need for transplantectomy, and allograft rejection. Comorbidity was determined by the age-adjusted Charlson Comorbidity Index (CCI), based on the previous medical history. The CCI is a weighted score which predicts the 1-year mortality of a patient, based on coexisting medical conditions and age [10].

For completeness, we added general questions to the enquiry to assess potential confounding factors. For example, 90 % of diabetic patients develop polyneuropathy at some time and this could lead to bias [11]. Furthermore, patients with chronic obstructive pulmonary disease are known to have a high incidence of chronic pain in general [12].

### Questionnaire

The questionnaire consisted of the Carolina Comfort Scale (9), the Visual Analog Scale (VAS-scale) [13], the Numeric Pain Rating Scale (NPRS-scale) [14], and a few questions aimed at their medical history and use of analgesics (Appendix). The CCS was originally validated for pain assessment after inguinal hernia repair with use of a mesh [9]. For this study, we modified the CCS to kidney transplantation in which the mesh-related questions were removed (Table 1). Instead, patients were asked if they experienced any inguinal pain or limited range of motion when certain activities are performed. After the first request to participate, all non-repliers were contacted by telephone in order to obtain the results. A previous study, which validated the questionnaires in a Dutch population of patients with inguinal hernia, reported 60 % response rate [9]. Because response rate in a population of chronic kidney disease is likely to be lower, the minimum response rate was set at 50 %. Chronic inguinal pain was defined as any patient who suffered from inguinal complaints ≥3 months after surgery, in accordance with hernia repair surgery [5]. Patients who scored a CCS ≥ 2 on one of the subcategories were considered as patients with significant and bothersome inguinal complaints.

**Table 1:** Modified Carolina Comfort Scale

*While lying down, do you have*							
Pain	0	1	2	3	4	5	n/a
*While bending over, do you have*							
Pain	0	1	2	3	4	5	n/a
Movement limitations	0	1	2	3	4	5	n/a
*While sitting up, do you have*							
Pain	0	1	2	3	4	5	n/a
Movement limitations	0	1	2	3	4	5	n/a
*While performing activities of daily life, do you have*							
Pain	0	1	2	3	4	5	n/a
Movement limitations	0	1	2	3	4	5	n/a
*While coughing or deep breathing, do you have*							
Pain	0	1	2	3	4	5	n/a
Movement limitations	0	1	2	3	4	5	n/a
*While walking, do you have*							
Pain	0	1	2	3	4	5	n/a
Movement limitations	0	1	2	3	4	5	n/a
*While walking up the stairs, do you have*							
Pain	0	1	2	3	4	5	n/a
Movement limitations	0	1	2	3	4	5	n/a
*While exercising, do you have*							
Pain	0	1	2	3	4	5	n/a
Movement limitations	0	1	2	3	4	5	n/a

*0* no symptoms, *1* mild but not bothersome symptoms, *2* mild and bothersome symptoms, *3* moderate and/or daily symptoms, *4* severe symptoms, and *5* disabling symptoms

### Statistical analysis

Categorical variables were analyzed by means of the *χ* ^2^ test or Fisher’s exact test and presented as numbers or percentages presented as mean ± standard deviation (SD). Continuous variables were tested with the Student’s *t* test for normal distribution and the Mann–Whitney *U* test for skewed distribution and presented as median ± interquartile range (IQR).

Linear regression method was used to perform univariate and multivariate analyses on factors associated with a higher score. Missing values were evaluated by Little’s MCAR test and, depending of level of significance, missing data were replaced by either expectation maximization technique (EM) or multiple imputation regression method (MI). Two-tailed P values were used throughout and significance was set at *p* < 0.05. All statistical analyses were done with the Statistical Package for the Social Sciences (SPSS 21.0, SPSS, Chicago, IL, USA, 2012).

## Results

From 2011 to 2013, 403 consecutive patients underwent kidney transplantation. Based on before-mentioned exclusion criteria, 58 patients were excluded due to death during follow-up (*n* = 24), median laparotomy (*n* = 12), incapability of reading Dutch or English (*n* = 6), mental retardation or neurological deficit (*n* = 11), and patients who underwent two kidney transplantations within the study period (*n* = 5). Of the 345 remaining patients, 199 patients returned the questionnaire (58 %) of which 82 were female (41 %) and 116 were male (59 %). The median follow-up was 22 months (IQR 12–30). In total, 86 % of participants completed the questionnaire resulting in 97 % of items being answered. In Table 2, the patient characteristics are shown.

**Table 2:** Patient characteristics

Variable	*N* patients
Age (years)	53 (SD 12.4)
*Sex*	
Male	117 (58.8 %)
Female	82 (41.2 %)
BMI	26.3 (SD 3.95) (Range 15.8–40.4)
Median ASA classification	3 (IQR 2–3)
Median CCI	4 (IQR 3–5)
*Cause of kidney failure*	
Polycystic kidney disease	43 (21.6 %)
(Hypertensive) nephrosclerosis/FSGS	35 (17.6 %)
IgA nephropathy/Henoch–Schönlein	27 (13.6 %)
Unknown origin	19 (9.5 %)
Autoimmune mediated	19 (9.5 %)
Diabetic nephropathy	15 (7.5 %)
Glomerulonephritis, pathy	11 (5.5 %)
Urological	9 (4.5 %)
Renal agenesis/renal atrophy	9 (4.5 %)
Vascular/ischemic	6 (3.0 %)
(Medical) drugs	6 (3.0 %)
*Pre-emptive transplantation*	
Yes	58 (29.1 %)
No	141 (70.9 %)
*Type of donor*	
Living related	55 (27.6 %)
Living unrelated	59 (29.6 %)
DCD	46 (23.1 %)
DBD	39 (19.6 %)
*Side donor nephrectomy*	
Right	66 (33.2 %)
Left	130 (65.3 %)
Unknown	3 (1.5 %)
*Fossa recipient*	
Right	160 (80.4 %)
Left	39 (19.6 %)
*Complications within 30 days*	111 (55.8 %)
Clavien-Dindo grade III–IV	63 (56.8 %)
Need for reoperation	11 (9.9 %)
DGF	54 (27.1 %)
Allograft rejection	35 (17.6 %)
Transplantectomy	4 (2.0 %)
Median follow-up in months	22 (IQR 12–30)

Patient characteristics*. BMI* body mass index, *ASA* American Society of Anaesthesiologists, *CCI* Charlson Comorbidity Index Score, *FSGS* focal segmental glomerulosclerosis, *DCD* donation after cardiac death, *DBD* donation after brain death, *DGF* delayed graft function, *Clavien-Dindo grade III*–*IV* any complication which requires surgical, endoscopic, or radiological intervention

### Outcomes Carolina Comfort Scale

**Fig. 3:**
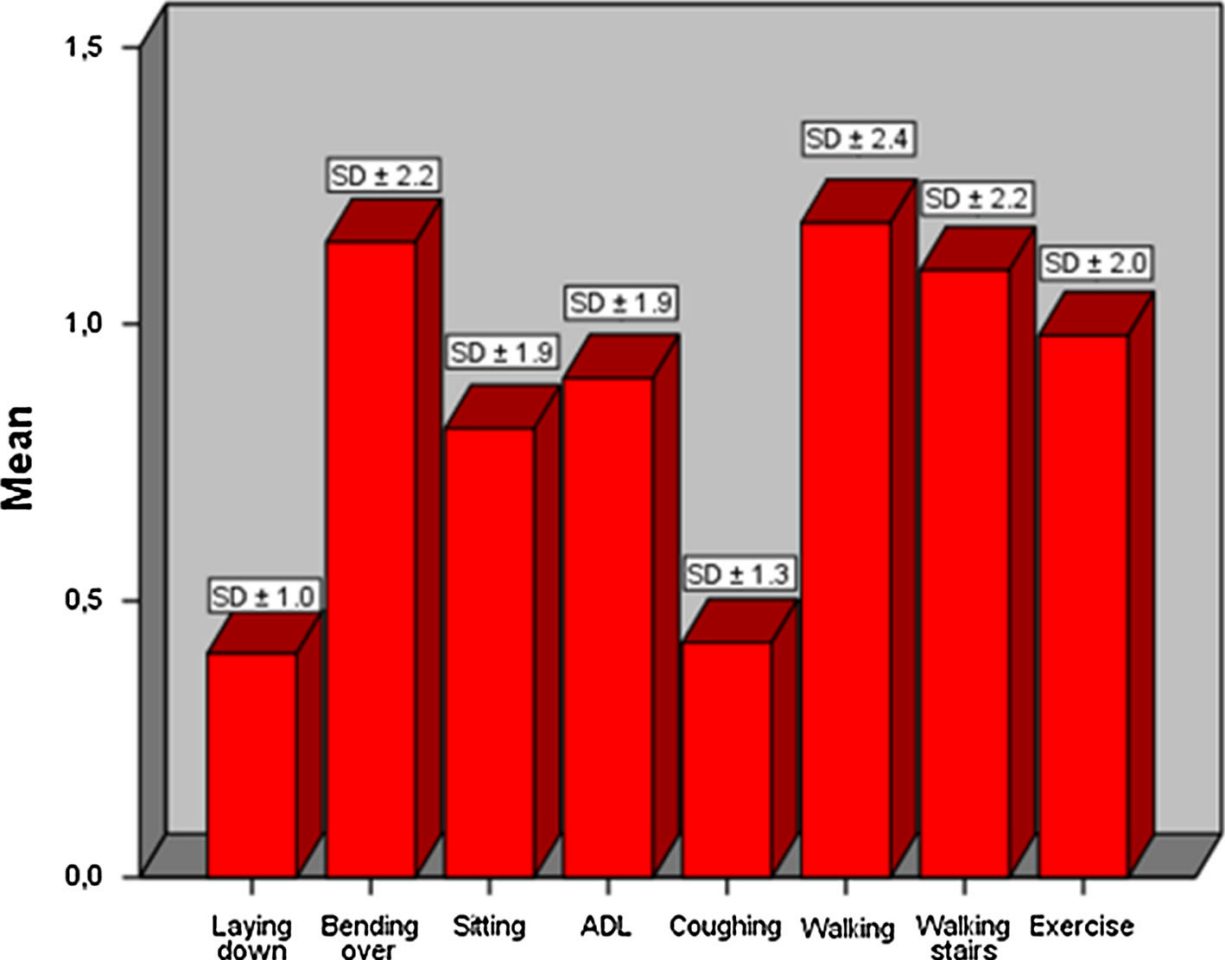
Mean outcomes of modified Carolina Comfort Scale for all eight subcategories

The mean score of all patients was 6.9 (SD ± 12.5, range 0–55). In total, 90 patients (45 %) reported a CCS > 0 and 64 patients (32 %) experienced bothersome complaints (CCS ≥ 2) on one or more subcategories of the CCS. Bothersome movement impairments occurred in 54 patients (27 %). Most inguinal complaints were reported during bending over and walking with a mean CCS score of 1.1 (SD ± 2.2) and 1.2 (SD ± 2.4), respectively. Mean outcomes are shown in Fig. 3.

Using univariate analysis, BMI (*p* = 0.01), need of a second operation (*p* = 0.038), and DGF (*p* = 0.033) were associated with a higher CCS score. In multivariate analysis, BMI (*p* = 0.02) remained an independent factor associated with a higher CCS score. (Table 3). The length of follow-up was not associated with a lower CCS score (*p* = 0.271).

**Table 3:** Univariate and multivariate analyses

	Lying down	Bending over	Sitting	ADL	Coughing	Walking	Walking stairs	Exercise	Total CCS
*Univariate analysis*									
BMI	0.088	**0.013**	**0.026**	0.139	**0.030**	**0.044**	**0.044**	**0.003**	**0.010**
DGF	0.102	0.088	**0.014**	**0.040**	0.284	**0.024**	**0.011**	0.864	**0.033**
Second OK	0.267	**0.013**	0.078	0.184	**0.001**	**0.035**	0.096	0.729	**0.038**
*Multivariate analysis*									
BMI	0.137	**0.024**	**0.041**	0.188	0.061	0.066	0.069	**0.003**	**0.010**
DGF	0.156	0.184	**0.022**	**0.040**	0.443	**0.024**	**0.011**	0.930	0.053
Second OK	0.418	**0.024**	0.172	0.254	**0.001**	0.056	0.151	0.971	0.066

Bold values are statistically significant

Univariate and multivariate analyses of variables associated with the different categories of the Carolina Comfort Scale (CCS)

*BMI* body mass index, *DGF* delayed graft function, *ADL* activities of daily life, and only BMI was considered as an independent factor associated with a higher CCS score

All patients were asked to answer additional questions concerning their comorbidity and use of analgesics. Eleven percent of patients had ipsilateral inguinal surgery prior to or after the kidney transplantation. The incidence rates of chronic obstructive pulmonary disease and diabetes was 6.5 and 27.6 %, respectively. About 50 % of the diabetic patients were new onset diabetes after transplantation (NODAT). Eleven percent of all patients used analgesics for inguinal complaints at time of completing the survey after a mean follow-up of 21.7 months (SD ± 11.3). Despite these possible confounding factors, no differences in incidence of chronic pain could be found during sensitivity analysis when excluding patients with diabetes, COPD, or prior inguinal surgery.

### VAS and NPRS scales

The mean VAS score was 10 (SD ± 20.1) and mean NPRS was 1.2/10 (SD ± 2.0).

Fifty-one percent of the patients had a VAS > 0 and 39 % had NPRS > 0 at the time of the survey. The age-adjusted CCI was associated with a higher VAS score (*p* = 0.007). DGF was associated with a higher NPRS score (*p* = 0.032) All other factors, including BMI, were nonsignificant in both scales.

## Discussion

This study shows that 32 % of patients suffer from chronic inguinal pain or impaired range of movement after kidney transplantation. The most important predictor of chronic inguinal pain after kidney transplantation in this study was BMI, which proved independent of known confounders as DM, COPD, and previous hernia surgery. Previous studies have reported a similar association between chronic pain and BMI after inguinal herniorrhaphy [15, 16]. This finding seems to indicate that inguinal herniorrhaphy and kidney transplantation have a similar etiology regarding chronic pain.

Identification of the nerves reduces the chance of iatrogenic damage and chronic pain. Also, when accidentally a nerve gets injured despite this nerve minded attitude it can still be recognized and a decent neurectomy can be performed to prevent a neurinoma. Previous studies have already shown that detection is feasible during inguinal herniorrhaphy when adequate anatomical knowledge is present, which provides opportunities during kidney transplantation [17, 18]. Inguinal hernia surgery has learned that regarding pain, preoperative identification of the nerves has better results than neglecting the nerves [19, 20]. But there is no evident difference between sparing of the nerves and standard neurectomy [21, 22]. However, considering the caused numbness identification is preferred. In kidney transplantation, the nerves will be identified more proximal, and therefore, neurectomy could additionally lead to motoric dysfunction of the oblique muscles of the abdominal wall [23]. Therefore, the authors advocate to spare the nerves after identification and only perform a neurectomy when there is suspicion of iatrogenic injury.

During kidney transplantation, no conscious detection of these nerves is generally performed, which may be an explanation for the high incidence of chronic inguinal pain. Whether detection of the nerves in kidney transplantation is sensible and wise cannot be concluded based on our results. Perhaps awareness of the etiology and incidence is a first step and additionally will result in better patient education. Given the risk in obese patients, an appropriate identification will be challenging. And, extending the duration of surgery will lead to a prolonged ischemia time with the associated risk of complications. However, with adequate anatomical knowledge the detection appears to be not time-consuming, and preventing pain will have a significant effect on retaining quality of life [17]. Therefore, we would carefully suggest that identifying the inguinal nerves should be performed during transplantation, especially in high-risk patients, and when perioperative iatrogenic damage is suspected, a surgical neurectomy should be considered.

A third of the patients with chronic inguinal pain do not receive any pain treatment which increases the need of healthcare services [3]. Our study showed a similar outcome, in which only 24 % of patients with inguinal complaints used targeted painkillers. This might be caused by the interactions with immunosuppressive therapies which often limit the use of specific analgesics. We believe that this under treatment could be prevented by a more intensive support from the primary treating physician in conjunction with a pain specialists and pharmacists. Furthermore, the use of regional nerve blockade could be an additional method of pain relief in the first hours/days after surgery [24].

The incidence of chronic pain in general in patients with end-stage renal disease is one of the highest of all chronic diseases and is reported up to 30–50 % of patients receiving hemodialysis [3]. After transplantation, this incidence will remain the same but the location seems to change, most likely caused by the immunosuppressive or antirejection therapy and the inguinal pain after the kidney transplantation. Previous studies have shown that 50 % of kidney recipients experience pain at more than one location [3, 25]. To assess this possible bias, we specifically asked all patients to describe the anatomical location where they experienced the most pain. In total, 118 of 199 (59 %) patients have answered this question and 53 of 118 (45 %) experienced the most pain in the inguinal region. When performing a sensitivity analysis with patients who experienced the most pain in inguinal region, both BMI as the need of re-operation were still significantly associated with a higher CCS score.

The relation between BMI and chronic pain is a widely known association and it seems to be a multifactorial phenomenon based on both psychosocial and biological factors [26]. One of the biological factors could be endocrine changes in fatty tissues which are characterized by a low-grade systemic inflammation. This could impair the pain modulation and lead to a lower pain threshold. The combination of these factors with surgical trauma and increased wound tension could be an explanation for increased chronic inguinal pain in obese patients.

This study has a few limitations that need to be addressed. First, about 11 % of patients underwent ipsilateral inguinal surgery prior or after transplantation. To correct for this confounder, we performed a sensitivity analysis in which we excluded these patients which yielded the same results. Secondly, the response rate was only 58 %. We are fully aware this could have led to selection bias. All non-responders were contacted by phone and reasons varied from recent illness to participation in many other studies. Absence of chronic inguinal pain was incidentally given as a reason not to participate. The study which validated the questionnaire in a rather healthy hernia population reported a 60 % response rate [9]. Therefore, we believe our study cohort is a good representative for the kidney recipients’ population, and the incidence of chronic pain is not overestimated.

Thirdly, because some patients already suffered from atypical chronic pain, this may have influenced our results. A previous study reported that the presence of preoperative pain is an independent factor for chronic pain after inguinal herniorrhaphy [27]. However, in our study the incidence of chronic inguinal pain in patients with pre-emptive transplantation was comparable with patients who already received some form of renal placement therapies (*p* = 0.631), and allograft rejection was not associated with a higher score.

A higher age-adjusted CCI score was associated with a higher VAS score and DGF was associated with a higher NPRS score. However, both scales are unidimensional and therefore do not seem the most suitable to use after kidney transplantation.

A new prospective study should be performed to assess whether nerve identification in kidney transplantation is feasible and leads to a decrease in chronic pain.

## Conclusion

Chronic inguinal neuropathic pain after kidney transplantation is common and seems to have a similar etiology as observed in inguinal hernia repair. Detection of the inguinal nerves is advised after hernia repair and leads to a decrease in chronic pain and improved quality of life. The same approach may be considered during kidney transplantation although it is unclear whether the same positive effects are obtained. This study intends to increase awareness among kidney transplantation surgeons in order to properly inform kidney recipient, especially obese patients, about the risk of chronic pain and consider appropriate measures.

## Abbreviations

CCS: Carolina Comfort Scale
VAS: Visual Analog Scale
IQR: Interquartile range
BMI: Body mass index
QoL: Quality of life
ASA: American Society of Anaesthesiologists
CCI: Charlson Comorbidity Index
NPRS: Numeric pain rating scale
SD: Standard deviation
DGF: Delayed graft function
NODAT: New onset diabetes after transplantation
DM: Diabetes mellitus
COPD: Chronic obstructive pulmonary disease
EM: Expectation maximization
MI: Multiple imputation
